# Identification and Validation of a Novel Biologics Target in Triple Negative Breast Cancer

**DOI:** 10.1038/s41598-019-51453-w

**Published:** 2019-10-17

**Authors:** Vikram B. Wali, Gauri A. Patwardhan, Vasiliki Pelekanou, Thomas Karn, Jian Cao, Alberto Ocana, Qin Yan, Bryce Nelson, Christos Hatzis, Lajos Pusztai

**Affiliations:** 1 Department of Internal Medicine, Section of Medical Oncology, Yale Cancer Center, Yale University School of Medicine, New Haven, CT USA; 2 Department of Pathology, Yale University School of Medicine, New Haven, CT USA; 3 Department of Obstetrics and Gynecology, Goethe-University Frankfurt, Frankfurt, Germany; 4 Department of Pharmacology, Yale Cancer Biology Institute, Yale University, New Haven, CT USA

**Keywords:** Breast cancer, Target identification, Breast cancer

## Abstract

The goal of this study was to identify a novel target for antibody-drug conjugate (ADC) development in triple negative breast cancer (TNBC), which has limited treatment options, using gene expression datasets and *in vitro* siRNA/CRISPR and *in vivo* functional assays. We analyzed 4467 breast cancers and identified GABRP as top expressed gene in TNBC with low expression in most normal tissues. GABRP protein was localized to cell membrane with broad range of receptors/cell (815–53,714) and expressed by nearly half of breast cancers tissues. GABRP gene knockdown inhibited TNBC cell growth and colony formation *in vitro* and growth of MDA-MB-468 xenografts in nude mice. Commercially available anti-GABRP antibody (5–100 μg/ml) or *de novo* generated Fabs (20 μg/ml) inhibited TNBC cell growth *in vitro*. The same antibody conjugated to mertansine (DM1) also showed significant anticancer activity at nanomolar concentrations. Our results indicate that GABRP is a potential novel therapeutic target for ADC development.

## Introduction

Triple negative breast cancers (TNBC) lack amplification of HER2 and expression of estrogen and progesterone receptors, and represent 15% of all breast cancers. Currently the only approved treatment options for newly diagnosed TNBC patients are chemotherapy agents^1,2^. Antibody drug conjugates (ADCs) allow targeted delivery of highly toxic agents to cancer cells that express a specific antigen without extensive adverse effects on normal tissues^3,4^. These drugs are emerging as a potential new strategy to treat TNBC. Preliminary results from clinical trials with IMMU-132 (Sacituzumab Govitecan, targeting TROP2 cell surface receptor), CDX-011 (Glembatumumab vedotin, targeting glycoprotein gpNMB overexpressed in many cancers), and SGN-LIV1A (that targets a zinc transporter SLC39A6) show around 30% objective tumor response rates in patients with metastatic TNBC that progressed on multiple prior lines of chemotherapies (NCT02161679, NCT01997333, NCT01969643). Our goal was to identify new targets for ADC development in TNBC. We compared mRNA expression profiles of TNBC with other breast cancer subtypes to identify genes that are overexpressed on TNBC surface, and identified gamma-amino-butyric-acid receptor π subunit (GABRP) as the most promising candidate gene for further functional experiments.

The GABRP gene encodes the π subunit of the gamma-aminobutyric acid (GABA) A receptor. The GABAA receptor is a heteropentameric, ligand-gated chloride channel composed of multiple subunits including α1–6, β1–3, γ1–3, δ, ε, θ, π and ρ1–3. Each subunit consists of a conserved extracellular domain, 4 transmembrane domains (TM1–4) and intracellular loops^5–7^. GABAA receptors in the central nervous system are most frequently made up of two α, two β and γ subunits^7^ and function as inhibitors of synaptic transmission. The function of the π subunit is not clear; it is not detectable in neuronal tissues and it is predominantly expressed at low levels in reproductive organs such as the uterus, ovary, prostate and breast^8,9^. GABRP levels change during the reproductive cycle and gestation and are upregulated during the window of implantation in the human endometrium, suggesting a role in these processes^10–12^. We have previously identified GABRP as a highly expressed gene in TNBC^13^. In this paper, we investigate the function of GABRP in TNBC growth *in vitro* and *in vivo* and assess its potential as a candidate for ADC development.

## Results

### GABRP mRNA is highly expressed in TNBC

**Figure 1:**
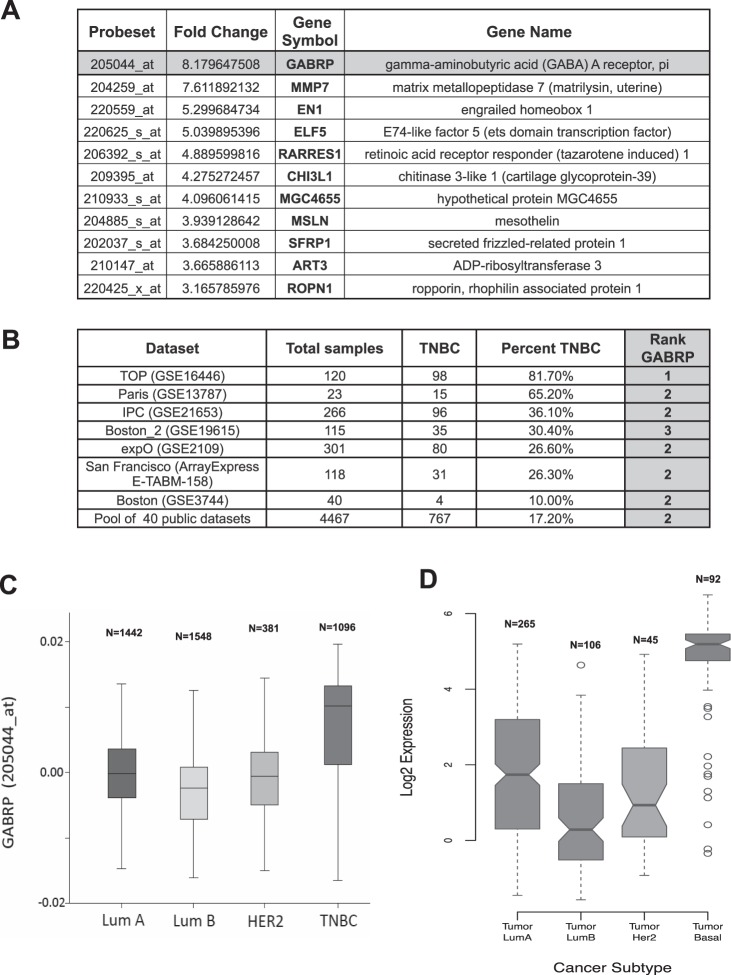
GABRP gene expression in breast cancer. (**A**) List of the top differentially overexpressed genes between triple-negative (n = 73) and receptor-positive (n = 221) breast cancers, sorted by fold-difference. This data set includes Affymetrix U133A gene expression data from 294 fine-needle aspiration (FNA) samples of stage I-III breast cancers obtained at MDACC. (**B**) We used seven independent Affymetrix datasets separately to validate the overexpression of the identified genes in TNBC. The probesets were ranked according to the mean expression difference between TNBC and non-TNBC samples separately in the seven datasets and additionally in a combined pool of all 40 data sets and GABRP ranked most consistently as one of the first three probesets in these lists. (**C**) GABRP gene expression in different molecular subtypes of breast cancer. Box plots of GABRP gene expression measured by Affymetrix microarray (probeset 205044_at) are shown for molecular subtypes of breast cancer defined by a single marker method according to Hugh *et al*. (Hugh *et al*., 2009) among 4467 pre-therapeutic invasive breast cancer samples from 40 datasets. (**D**) GABRP gene expression in the intrinsic breast cancer subtypes as defined in The Cancer Genome Atlas (TCGA) dataset.

We identified 681 Affymetrix U133A gene chip probe sets with at least two-fold overexpression in TNBC versus non-TNBC with false discovery rate (FDR) < 0.0001 observed in two independent datasets. GABRP displayed the highest fold-change (8.18×) in the MDACC cohort (Fig. 1A, Supplementary Table 1), the second highest fold-change (12.76×) in the Wang cohort (Supplementary Table 1), and ranked consistently among the top three probe sets in each validation dataset (Fig. 1B, Supplementary Tables 1, 2) and in the pooled validation cohort (Fig. 1C**)**. GABRP was also overexpressed in the basal subtype of TCGA breast cancer cohort (Fig. 1D). All expression data, subtype designations and links to datasets are in Supplementary Table 3.

Since target expression in normal tissues is a potential indicator of systemic toxicity, we examined GABRP mRNA expression in normal and cancer tissues and compared with targets of ADCs already showing success in the clinic (Supplementary Fig. 1). GABRP expression in normal tissues was low and comparable to that of ERBB2 (the target of TDM1, Kadcyla^TM^) and gpNMB (Supplementary Fig. 1). We also observed high expression of GABRP in subsets of gastric and colorectal cancers and lung adenocarcinoma suggesting a broader potential therapeutic appeal.

We also examined GABRP mRNA expression in breast cancer cell lines in the Cancer Cell Line Encyclopedia (CCLE) (Supplementary Fig. 2). In contrast to clinical datasets, GABRP transcript levels were comparable between most TNBC and non-TNBC breast cancer cell lines. Such variation between transcripts in cell lines versus clinical tissues may result due to adaptation of cell lines to growing in highly artificial nutritional and microenvironment of plastic dishes.

### GABRP protein is predominantly localized to the membrane

**Figure 2:**
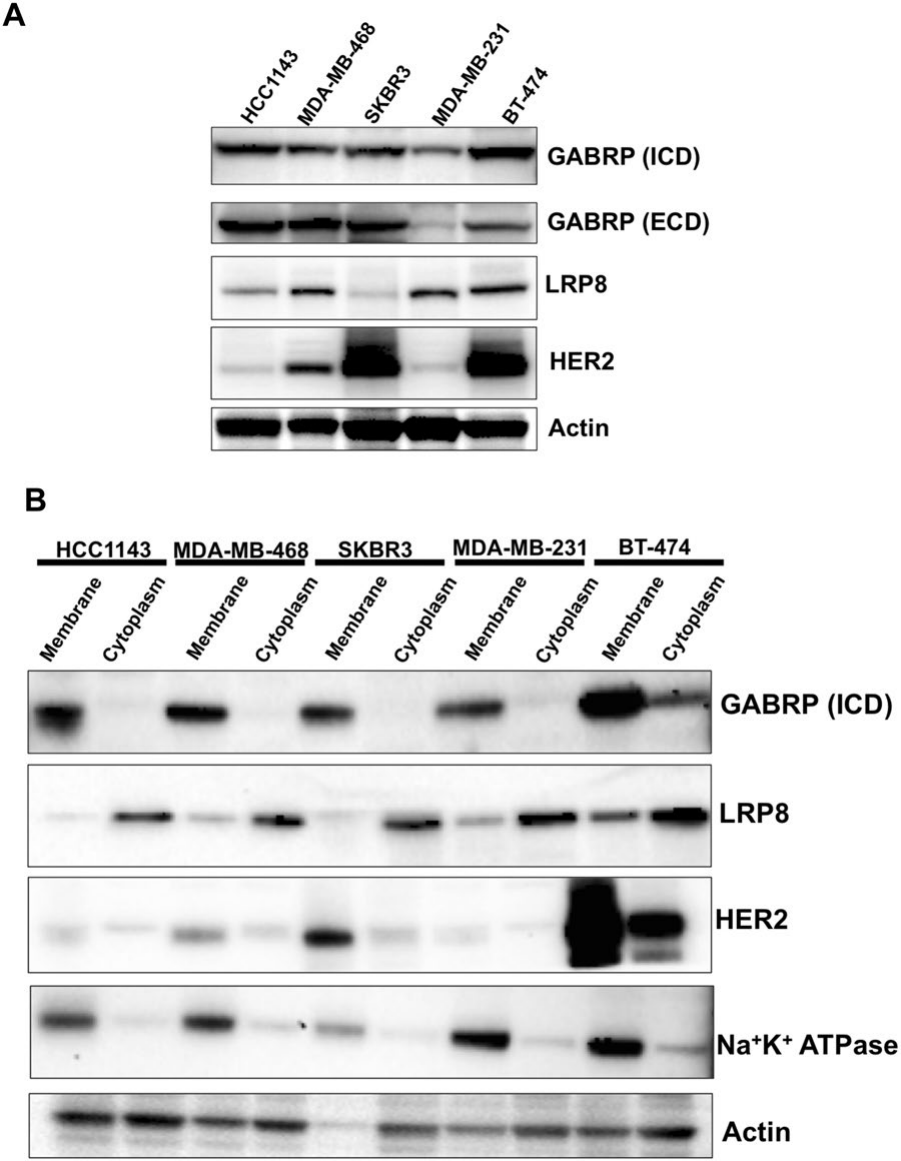
GABRP protein expression in breast cancer cell lines. (**A**) GABRP protein was detected in Western blots using intracellular domain (ICD) and extracellular domain (ECD) binding GABRP antibodies. LRP8, another potential target overexpressed in TNBC, was also assessed. HER2 served as a control for HER2 amplified cell lines (SKBR3 and BT-474), while actin was used as a loading control. (**B**) Membrane and cytoplasmic protein extracts were obtained by subcellular protein fractionation. Western blot analysis was performed to determine GABRP levels using these subcellular protein fractions of each cell line. Na^+^K^+^ATPase was used as a positive control for membrane protein, while actin was used as a loading control.

We examined GABRP protein levels in five breast cancer cell lines and similar to the mRNA expression data, we found that both TNBC (HCC1143, MDA-MB-468, MDA-MB-231) and non-TNBC (SKBR3 and BT-474) cells express comparable GABRP protein levels (Fig. 2A). This observation was confirmed using two distinct anti-GABRP antibodies targeting the intracellular (ICD) and extracellular (ECD) domains, respectively. We assessed the protein levels of low density lipoprotein receptor-related protein 8 (LRP8), another highly expressed gene in TNBC that we also considered as potential ADC target (Supplementary Table 1, Fig. 2A). LRP8 belongs to low-density lipoprotein (LDL) receptor family involved in homeostatic management of lipid and cholesterol trafficking in human body. We found LRP8 as one of the overexpressed genes in TNBC and also one of the top hits from our siRNA screen across 18 breast cancer cell lines, showing significant and preferential growth inhibition in TNBC cell lines (data not shown). LRP8 was located mainly in cytoplasm and was not investigated further; GABRP was predominantly localized in the membrane fraction (Fig. 2B).

### The number of GABRP receptors on cell surface is similar to other ADC targets

**Figure 3:**
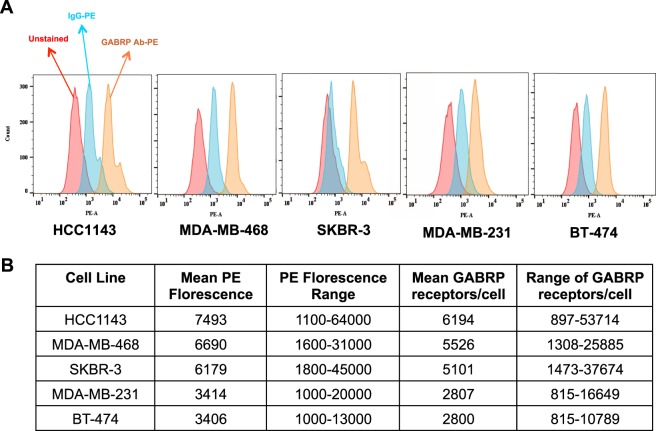
GABRP quantification on cell surface. The number of GABRP receptors on the cell surface was quantified by BD QuantiBRITE PE fluorescence quantitation kit for flow cytometric analysis (BD Biosciences). (**A**) ECD-binding GABRP and IgG antibodies conjugated to phycoerythrin (PE) were used for the flow cytometric analysis. (**B**) Mean number of GABRP receptors on the cell surface was estimated by PE florescence intensity, as antibody binding capacity (ABC). QuantiBRITE beads labeled with different PE levels were used to generate the standard curve for florescent intensity versus the number of PE molecules/bead.

We quantified GABRP density on cell surface using QuantiBRITE PE flowcytometry assay (Fig. 3A). Receptor numbers ranged between 815–53,714 receptors/cell with large variation within each cell line. Mean receptor numbers per cell line ranged between 2,800–6,194 (Fig. 3B). These expression levels are within the range of receptor numbers that have been targeted successfully in the clinic^14–21^. However, clinical response to ADC is determined by multiple factors including receptor turnover rate, inflammatory and immune response, and by-stander effects of the toxins released, in addition to surface receptor density.

### GABRP protein expression in tumor tissues by immunofluorescence (IF) and immunohistochemistry (IHC)

**Figure 4:**
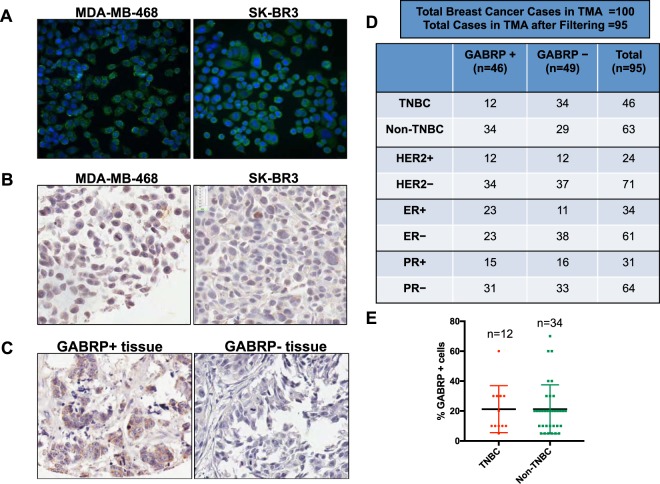
GABRP protein detection by IF and IHC in breast cancer cell lines and breast tumors. (**A**) IF detection of GABRP (green) in MDA-MB-468 (left) and SK-BR3 (right) showed membrane and cytoplasmic localization of the protein. Magnification 20×. Cell nuclei were counter-stained with Hoechst (blue) dye. (**B**) GABRP detection by chromogenic IHC in FFPE TNBC cell pellets (MDA-MB-468, left) and (SK-BR3, right) from the Yale breast cancer index tissue microarray (YTMA279) showing cytoplasmic and membranous localization of the protein. (**C**) Representative images from IHC chromogenic detection of GABRP in breast cancer TMAs. GABRP-positive case (left) with signal in tumor epithelial cells and a GABRP-negative case (right). Magnification 40X. (**D**) Distribution of GABRP-positive and negative cases in the breast cancer TMA stained by IHC. Minimum of 100 tumor epithelial cells within each case were examined and positivity cut-off was set at 1%. (**E**) Comparison of the percentage of GABRP positive cells in TNBC and non-TNBC tumors in the cases scored positive from breast cancer TMAs.

We next examined GABRP protein with IF (Fig. 4A, Supplementary Fig. 4), and chromogenic IHC of FFPE sections of cell line pellets (Fig. 4B) and tissues of surgically resected primary breast cancers (Fig. 4C). The FFPE tissues included 100 cancer cases (one core per case) on a tissue microarray (Fig. 4C). Out of 95 evaluable cases, 46 scored positive for GABRP expression. Interestingly, GABRP positive cases were also found among hormone receptor positive breast cancers (Fig. 4D). Overall, 12/32 (37%) of TNBC cases and 34/63 (54%) of non-TNBC cases were positive for GABRP protein expression using a 1% threshold to define positivity (Fig. 4D) with no significant difference in percentage of GABRP tumor cells in GABRP-positive TNBC and non-TNBC tumors (Fig. 4E). Within each breast cancer subtype, GABRP expression was heterogeneous and mainly detected in tumor epithelial cells.

### GABRP is critical for TNBC cell growth *in vitro* and *in vivo*

**Figure 5:**
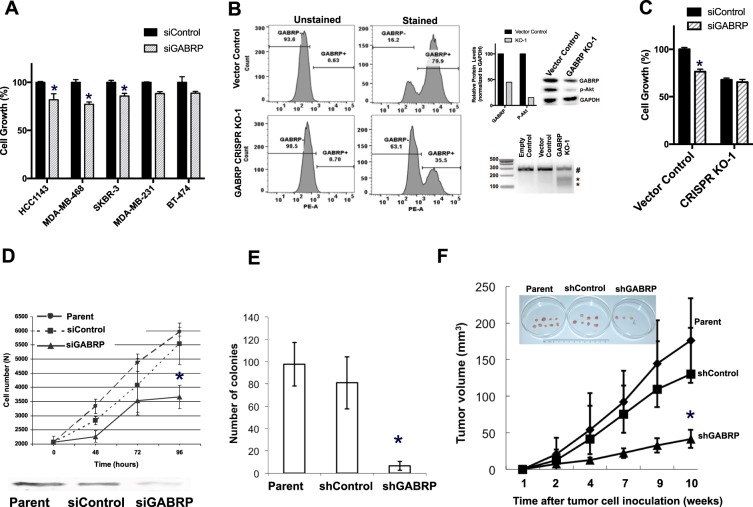
Effect of GABRP knock-down *in vitro* and *in vivo*. (**A**) Proliferation of 50 nM scrambled control siRNA (siControl) or GABRP siRNA (siGABRP) treated cells was assessed by CellTiter Glo luminescent viability assay after 72 hours and indicated as percentage of respective control for each cell line. Each vertical bar represents the percent cell growth measured as mean luminescence ± SEM in each treatment group. *P < 0.05 as compared to respective siRNA control group. (**B**) GABRP was assessed in vector control and GABRP CRISPR KO MDA-MB-468 (KO-1) cells using FLAG tagged ECD-binding monoclonal Fab and anti-FLAG-PE antibody by flow-cytometry. GABRP protein expression was assessed by immunoblotting and densitometric analysis was performed in ImageJ software. Empty control (no guide sequence), Vector control (scrambled sgRNA sequence), and GABRP KO-1 MDA-MB-468 cells were harvested for genomic DNA isolation and analyzed by T7 endonuclease assay. Pound sign (#) indicated undigested DNA and stars (*) indicate digested products. (**C**) Proliferation of 50 nM scrambled control siRNA (siControl) or GABRP siRNA (siGABRP) treated vector control and CRISPR KO-1 cells was assessed by CellTiter Glo luminescent viability assay after 72 hours and indicated as percentage of siRNA control-treated vector control MDA-MB-468 cells. Vertical bar represents the percent cell growth measured as mean luminescence ± SEM in each treatment group. *P < 0.05 as compared to siRNA control-treated vector control MDA-MB-468 cells. **D**. Proliferation of control siRNA (siControl) or GABRP siRNA (siGABRP) treated or untreated MDA-MB-468 (parent) cells was assessed by counting cells after trypan blue exclusion assay over 96 hours; data points represent mean viable count per well ± SD in each group. Cells from these treatment groups were collected 96 h after transfection and probed for GABRP protein levels by Western blotting (below). (**E**) The stable GABRP knockdown cell line (shGABRP), shRNA control (shControl) and parent MDA-MB-468 cell lines were allowed to form colonies in soft agar. Vertical bars indicate average number of colonies in three replicates compared to parent cell line. *P-*values were calculated by Student *t* test. *P < 0.05. (**F**) Tumor volume of stable GABRP knockdown (shGABRP), stable control (shControl) and untreated MDA-MB-468 (parent) xenograft in female athymic Nude-Foxn1 nude mice (Nu/Nu) in (N = 5 in each group). *P-*values were calculated by Student *t* test. *P < 0.05. Tumors shown below were isolated at the end 10-weeks inoculation period.

To examine the effect of GABRP expression on tumor growth, we knocked down GABRP expression in five cell lines, and overexpressed it in GABRP-low MDA-MB-231 cell line. GABRP knock-down resulted in modest but significant suppression of growth in 72 h in high-GABRP TNBC cell lines-HCC1143 and MDA-MB-468, but not in low-GABRP MDA-MB-231 (Fig. 5A). In contrast, MDA-MB-231 cells overexpressing GABRP grow significantly faster than vector control cells (Supplementary Fig. 5). To assess specificity, we developed GABRP CRISPR KO HCC1143 and MDA-MB-468 cell lines, but a complete GABRP KO was not achieved in the polyclones. Figure 5B shows the decrease in GABRP expressing cells from nearly 80% in vector control to 35% in GABRP KO-1 MDA-MB-468 cells by flowcytometry, and 55% decrease in protein level by immunoblotting. This knockout efficiency was consistent with the efficacy of CRISPR sgRNA as determined by T7 nuclease assay showing digested DNA in knockout cells (Fig. 5B). While vector control MDA-MB-468 cells were sensitive, GABRP KO cells were resistant to GABRP knockdown (Fig. 5C). As expected, CRISPR-KO cells grew slower compared to the vector control cells. In a time-course experiment, we demonstrated greater growth suppression at 96 h after siRNA transfection in MDA-MB-468 cells (Fig. 5D). Additionally, we knocked down GABRP expression stably using shRNA in MDA-MB-468 TNBC cells. Soft-agar assay revealed that stable GABRP knockdown significantly decreased anchorage-independent growth over 3 weeks compared to control shRNA or untreated parent cells (Fig. 5E). Consistent with the *in vitro* results, we observed significantly reduced tumor growth and tumor formation in the knockdown cells compared to shRNA control or the parent MDA-MB-468 cells in female athymic Nude-Foxn1 mice (Fig. 5F). We have noted a decline in activated AKT levels in GABRP knockout cells, but whether these effects involve GABA_A_receptor complex or interplay of GABRP with other surface receptors, needs further investigation. Nevertheless, these results demonstrate an important role for GABRP in sustaining tumor growth both *in vitro* and *in vivo*, which is an advantage for a potential ADC target. Functionally vital targets carry lower risk for tumor downregulating the protein as a resistance mechanism. Another advantage is the likely potentiated response achieved due to blockade of receptor function in addition to toxin delivery.

### ADC generation

We commissioned ADCs from Levena Biopharma and requested conjugation of DM1 using a stable linker to (i) commercially available ECD bindingpolyclonal GABRP-antibody (GABRP-Ab) and (ii) polyclonal isotype control IgG. Excess DM1 was removed after conjugation and free DM1 was limited to <5% in each ADC batch by centrifugal ultrafiltration using 50 kDa molecular weight cut-off (MWCO) Amicon columns (7x rounds) and size exclusion chromatography (SEC). Unconjugated antibody was 5% for each ADC, as determined by hydrophobic interaction chromatography (HIC)-HPLC. Drug to antibody ratios (DAR) calculated using ultraviolet-visible spectrophotometry **(**UV-Vis) were 3.80 and 4.10 respectively.

### ECD binding anti-GABRP antibody and ADC suppresses tumor cell growth

**Figure 6:**
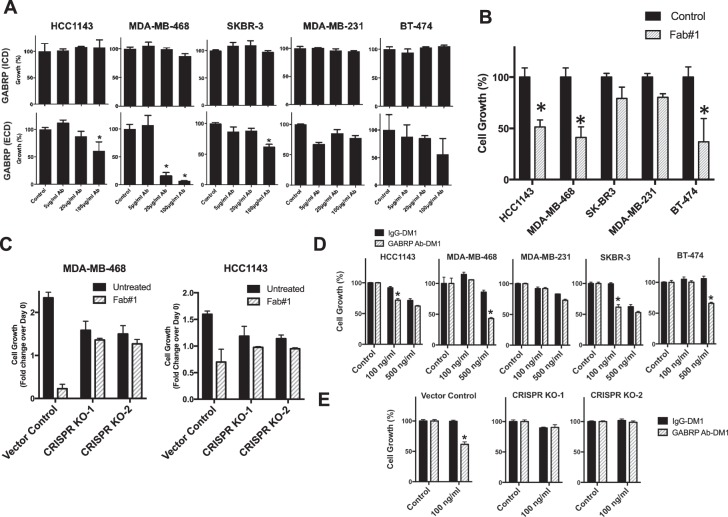
Effect of naked GABRP antibody and GABRP antibody-DM1 ADC. (**A**) GABRP antibody binding either ICD or ECD of GABRP were tested in culture for anticancer activity. Cells were seeded at 2000 cells/well in duplicate in 96-well plates and cell proliferation was assessed by the CellTiter-Glo luminescent assay at the end of 3-day culture period. Vertical bars indicate mean percent growth calculated as mean luminescence normalized to respective control ± SEM for each cell line. *P* values were calculated by Student *t* test. *P < 0.05. (**B**) GABRP ECD binding specific monoclonal Fab#1 (20 μg/ml) was tested in culture for anticancer activity. Cells were seeded at 2000 cells/well in duplicate in 96-well plates and cell proliferation was assessed by the CellTiter-Glo luminescent assay at the end of 3-day culture period. Vertical bars indicate mean percent growth calculated as mean luminescence normalized to respective control ± SEM for each cell line. *P-*values were calculated by Student *t* test. *P < 0.05. (**C**) Effect of Fab#1 was evaluated in CRISPR knock-out (KO-1 and KO-2) MDA-MB-468 and HCC1143 cell lines. Cells were seeded at 2000 cells/well in duplicate in 96-well plates and cell proliferation was assessed by the CellTiter-Glo luminescent assay at the end of 3-day culture period. Vertical bars indicate cell growth as fold-change in mean luminescence over Day 0, normalized to untreated vector control ± SEM. *P-*values were calculated by Student *t* test. *P < 0.05. (**D**) Cells were plated at 2000 cells/well in triplicate in 96-well plates and exposed to GABRP Ab-DM1 ADC and control IgG-DM1 for 3 days. Cell proliferation was similarly assessed by the CellTiter-Glo luminescent assay, and indicated by vertical bars as mean percent growth ± SEM, as compared with respective untreated control for each cell line. *P* values were calculated by Student *t* test. *P < 0.05. (**E**) Effect of GABRP Ab-DM1 ADC was evaluated in CRISPR knock-out (KO-1 and KO-2) MDA-MB-468 cells. Cells were seeded at 2000 cells/well in triplicate in 96-well plates and cell proliferation was assessed by the CellTiter-Glo luminescent assay after 48 h. Vertical bars indicate mean percent growth calculated as mean luminescence normalized to respective untreated control ± SEM for each cell line. *P* values were calculated by Student *t* test. *P < 0.05.

Some antibodies used in the clinic have anticancer activity even in the absence of conjugation to a toxic cargo^22^. We therefore, tested the naked (unconjugated) commercially available ECD-binding GABRP-Ab that suppressed the growth of breast cancer cell lines, particularly MDA-MB-468, in a dose-dependent manner starting at 20 μg/ml. The ICD-binding GABRP-Ab had no effect in concentrations up to 100 μg/ml (Fig. 6A). Next, we tested anti-GABRP monoclonal Fab (20 μg/ml) that we have generated against the ECD of GABRP and found even more pronounced growth inhibition **(**Fig. 6B**)** in the same cell lines. These effects are specific as CRISPR KO cell lines are more refractory to anti-GABRP Fab induced growth inhibition **(**Fig. 6C).

The anti-GABRP antibody-DM1 conjugate (GABRP Ab-DM1) showed significantly higher growth inhibitory effect at nanomolar concentrations (10–500) than control (IgG)-DM1 (Fig. 6D). Interestingly, ADC significantly suppressed growth in vector control but not in GABRP KO CRISPR KO cells **(**Fig. 6E**)**. These effects of anti-GABRP-DM1 were significant but modest indicating that the antibody and the conjugation technique will require further optimization^23^.

## Discussion

Our results suggest that GABRP is a potential novel therapeutic target in TNBC. Its membrane localization, high expression in breast cancers, low expression in most normal tissues and important role to sustain cell growth makes it a candidate for ADC development. We also provide proof-of-concept experiments demonstrating anticancer activity of a commercial naked anti-GABRP antibody, anti-GABRP Fab, and a DM1 conjugated ADC version of the same commercial antibody.

The distribution of GABRP mRNA expression across many cancer types suggests that an anti-GABRP-ADC may have therapeutic activity in subsets of several currently difficult-to-treat cancers including gastric, colorectal and lung. The generally low expression in normal tissues is reassuring, but we noted relatively high mRNA expression in the upper airways and lungs, although expression levels were still lower than seen in breast cancer (Supplementary Fig. 1). Similar high level of gpNMB mRNA expression in normal lung tissues can also been seen yet pulmonary toxicity was not observed with the anti-gpNMB ADC (Glembatumumab vedotin). ADC activity, to some extent, is proportional to the number of target molecules on the cell surface, however, the minimum number of cell surface molecules required for ADC effect varies and ranges between 1000 to >100,000 receptors/cell. We estimated the number of GABRP molecules to be 815–53,714 with broad variations across individual cells even within the same cell line. The mean receptor numbers per cell line ranged between 2,800 and 6,194 which is within the range of clinically useful ADCs: 4,695 for CD22, the target of approved ADC inotuzumab ozogamicin^19^; 1000–10,000 for CD33, the target of gemtuzumab ozogamicin^15,21^; 1000–9000 for CD54, the target of the therapeutic antibody BI-505, on the surface of myeloma cells^18,19^.

We also developed a potential IHC assay to assess GABRP protein expression in clinical tissues, and found that nearly half of all breast cancers were positive for GABRP, including ER positive cancers. This result was unexpected because the GABRP mRNA levels are significantly lower in ER positive cancers. It is important to recognize that the sensitivity of IHC and mRNA expression measurements to quantify gene expression are very different and occasional substantial discordance in mRNA and protein levels are well documented in the literature for several genes.

Antitumor activity of naked antibody is not a prerequisite for successful ADC development, as naked antibodies against targets such as CD30, CD138, SLC39A6, gpNMB alone do not show significant antitumor activity, despite substantial clinical activity of the conjugated antibodies. However, several successful ADCs’ targets are important for cell survival and therefore the naked antibody also has clinical activity (e.g. trastuzumab). We demonstrated in siRNA and CRISPR knockout experiments that GABRP expression is important for TNBC cell growth, both *in vitro* and *in vivo*, consistent with a previous *in vitro* report^24^. Somewhat surprisingly, we observed significant growth inhibitory effect of the naked polyclonal ECD-binding antibody, which implies that a significant fraction of the antibodies included in the polyclonal mix may not be binding to the same epitope, and therefore an optimized monoclonal antibody could produce even greater growth suppression. Therefore, we generated monoclonal anti-GABRP antibodies and Fabs by mouse immunization and by phage-display library screening, respectively, against the entire GABRP ECD as an antigen. Both mouse derived monoclonal antibodies (data not shown) and synthetically generated Fabs induced greater growth inhibition than the commercially available polyclonal antibodies.

To further credential GABRP as a potential therapeutic target, we used a commercially available anti-GABRP ECD-antibody for ADC generation and tested its effect on cell growth *in vitro*. We observed enhanced inhibitory activity compared to the isotype-matched control in GABRP expressing cells and not in GABRP KO isogenic background. The relatively low activity of our ADCs may be due to less than optimal internalization of the IgG and non-uniformity of DM1 conjugation. Using our method of conjugation, DM1 can bind to any of ~30 available IgG lysine sites and therefore the resulting ADCs are a mixture composed of variable number of toxic cargo on each antibody. Higher than 3/4 DM1 molecules conjugated to an antibody can reduce the antigen-binding potency, solubility and stability of an ADC^25,26^. The observed cytotoxicity of the isotype control-DM1 conjugates at higher concentrations is likely due to non-specific antibody internalization or free DM1 in the medium. These results support the target function of GABRP but also highlight the need for further antibody and conjugation optimization^23^.

Our systematic evaluation for target identification and functional validation followed by generation of specific GABRP Fabs and ADCs helped building a robust preclinical package for drug development. These steps provide a framework that can be extended to the discovery of other novel ADC targets, and in other cancer types.

## Methods

### Discovery and validation datasets

Affymetrix U133A gene expression profiles from 294 fine-needle aspiration (FNA) biopsies of stage I-III breast cancers at MD Anderson Cancer Center (MDACC)^27,28^ were used as the discovery cohort to define candidate TNBC-genes. A previously described gene expression dataset (Affymetrix U133A or U133Plus2.0 arrays) of 4467 breast cancer patients compiled from 40 publicly available data sets^29,30^ was used for validation. Additional details are in the Supplementary Methods.

### Cell lines

TNBC (HCC1143, MDA-MB-468, and MDA-MB-231) and non-TNBC HER2-amplified (SKBR-3 and BT-474) cell lines were purchased from the American Type Culture Collection (Manassas, VA) where all cell lines were authenticated by short tandem repeat profiling, karyotyping, morphology and cytochrome C oxidase I testing. Cell lines were obtained between 2011 and 2016, and used at passages 3–9, and cultured less than 3 months after resuscitation. Additional details are in the Supplementary Methods.

### Immunoblotting

Cells were plated at 1 × 10^6^cells/100 mm plate and grown to subconfluency, followed by extraction by RIPA buffer containing proteinase and phosphatase inhibitors for whole cell lysates. Membrane and cytoplasmic protein fractions were extracted using Subcellular Protein Fractionation Kit (Thermo Scientific). Lysate samples were electrophoresed in 4–12% NuPAGE SDS-polyacrylamide midigels (Life Technologies Corporation) and transblotted onto PVDF membrane as described previously^31^. For details, see Supplementary Methods.

### Flow-cytometry

Number of GABRP receptors on cell surface was quantified by BD QuantiBRITE PE (BD BioSciences) flow-cytometric analysis. GABRP ECD-binding specific monoclonal antibody-fragments (Fabs) were used in GABRP detection on cell surface in Fig. 5 and Supplementary 5. For details, see Supplementary Methods.

### Immunofluorescence (IF)

Cells grown to subconfluency in 24-well culture plates were fixed with 4% formaldehyde, permeabilized with 0.5% triton-X, blocked by 1% BSA and incubated overnight with primary antibody against GABRP (ICD binding), followed by staining with rabbit secondary antibody-tagged with AlexaFlor488 fluorochrome (green). Cell nuclei were counter-stained with Hoechst (blue) dye. Fluorescence was visualized under confocal microscope and 20X images were captured using imaging software (Perkin Elmer).

### Chromogenic immunohistochemistry (IHC)

GABRP IHC was performed on 5-μm whole tissue-sections of formalin-fixed paraffin embedded (FFPE) breast cancer tissue microarray (TMA, US Biomax, Inc.) This TMA containing 100 invasive breast cancer tissues with annotated clinical stage, pathology grade, IHC markers (ER, PR, HER2) status, was stained using GABRP antibody (Santa Cruz Biotech). For details, see Supplementary Methods.

### GABRP knock-down

Human GABRP siRNA oligonucleotides were purchased from Ambion (Austin, TX). For stable knockdown, we produced GABRP shRNA encoding pRETRO-SUPER-GABRP plasmid. For details, see **Supplementary Methods**. Knockdown of GABRP was also achieved by ~60% efficiency of GABRP CRISPR knockout (KO) in cell lines (polyclones); GABRP knockout (KO-1 and KO-2) MDA-MB-468 and HCC1143 cell lines were generated according to protocol described previously^32^. Surveyor T7 endonuclease assays were conducted as described previously^33^. Primer sequences and protocol details are in Supplementary Methods.

### Soft-agar assay

Soft agar plates were photographed and colonies were enumerated using ImageJ software version 1.46r.

### Mice xenografts

Female athymic Nude-Foxn1 nude mice (Nu/Nu) (Harlan) were housed under specific pathogen-free conditions, at The University of Texas MDACC approved by the American Association for Accreditation of Laboratory Animal Care in accordance with the current regulations and standards of the U.S. Department of Agriculture, Department of Health and Human Services, and National Institutes of Health. Experimental details are in the Supplementary Methods.

### Proliferation assay

Cell lines were plated at 2000 cells/well (3 wells/group) in clear bottom opaque-walled 96-well culture plates (Thermo Scientific) in media containing 5% FBS, and exposed to respective experimental treatments and counted in a hemocytometer after staining by 0.4% trypan blue or luminescence measured using the ATP-based CellTiter-Glo® luminescent cell-viability assay (Promega).

### GABRP antibody-DM1 ADC

To generate a novel ADC against GABRP, commercially available ECD binding GABRP antibody (Abcam) was conjugated to mertansine (DM1), a potent thiol-containing maytansinoid toxin, by ‘Immunogen Conjugation’ method to achieve drug to antibody ratio (DAR) of 3–4 (Levena BioPharma US, Inc). Further details are in the Supplementary Methods.

## Supplementary information

Supplementary Information

Supplementary Table 1

Supplementary Table 3

## Data Availability

All the data supporting the findings of this study are available within the article and its supplementary data files, and from the corresponding authors on reasonable request.
