# Supplementary material for: Identification and Validation of a Novel Biologics Target in Triple Negative Breast Cancer
Source: Sci Rep. 2019 Oct 17;9:14934. doi: 10.1038/s41598-019-51453-w (PMC6797726; doi:10.1038/s41598-019-51453-w)
Supplement: Supplementary file 1 — Supplementary Information [file 41598_2019_51453_MOESM1_ESM.pdf]

Identification and Validation of a Novel Biologics Target in Triple Negative Breast Cancer

Vikram B. Wali[1], Gauri A. Patwardhan[1], Vasiliki Pelekanou[2], Thomas Karn[3], Jian Cao[2], Alberto Ocana[1], Qin Yan[2], Bryce Nelson[4], Christos Hatzis[1], and Lajos Pusztai[1]

[1]Department of Internal Medicine, Section of Medical Oncology, Yale Cancer Center, Yale University School of Medicine, New Haven, CT, USA

[2]Department of Pathology, Yale University School of Medicine, New Haven, CT, USA

[3]Department of Obstetrics and Gynecology, Goethe-University Frankfurt, Frankfurt, Germany

[4]Department of Pharmacology, Yale Cancer Biology Institute, Yale University, New Haven, CT, USA

Corresponding author:

Vikram B. Wali, Ph.D. or Lajos Pusztai, Ph.D.
Department of Internal Medicine
Breast Medical Oncology
Yale Cancer Center
Yale University School of Medicine
300 George Street
New Haven, CT 06511

walivik@gmail.com

or

lajos.pusztai@yale.edu

Supplementary Methods

Differential gene expression in TNBC and non-TNBC datasets To identify genes that are highly expressed on TNBC cell surface, we used two independent data sets (MDACC and Wang cohorts) to define genes overexpressed in ER/PR- and HER2-negative cancers. In MDACC cohort, probe sets were tested for differential expression in TNBC (n=73) and non-TNBC cases (n=221) using an unequal variance t-test. A second dataset obtained from frozen tissues of surgically resected breast cancer specimens from 286 lymph-node-negative patients including 56 TNBC and 230 receptor-positive cases (Wang et al. 2005) was used to confirm overexpression of genes in TNBC. We focused on the 1871 genes that are overexpressed in TNBC vs non-TNBC at an FDR level of <0.00001. Sixty-two percent of these genes (n=1162) were also overexpressed in TNBC cases in second (Wang) independent human breast cancer dataset (individual P-values <0.05). Out of these, we discovered 681 genes that have at least two-fold overexpression in TNBC with p<0.0001 observed in independent datasets (Supplementary Table 1). To account for multiple comparisons we performed beta uniform mixture analysis (BUM) of the p values, that showed a non-uniform distribution and was used to calculate false discovery rates (FDR) for particular p-values.

Validation Cohorts Affymetrix CEL files were processed with the MAS5.0 algorithm of the *affy* package (Gautier et al., 2004) of the Bioconductor software project (Gentleman et al., 2004). Data from each array were $\log_2$-transformed, median-centered, and expression values of all the probesets from the U133A array were multiplied by a scale factor S so that the magnitude (sum of the squares of the values) equals one. The bimodal distributions of ESR1, PgR, and HER2 gene expression were used to derive cutoffs to differentiate high and low expression, or positive and negative status, respectively, as described previously (Karn et al., 2010). We used seven of the independent Affymetrix data sets each containing at least 10% TNBC samples separately to validate the overexpression of the identified genes

in TNBC. Assignment of intrinsic subtypes was based on the expression levels of ESR1, PgR, HER2 and Ki67, as previously described (Hugh et al., 2009). For a distinction of Luminal A and Luminal B subgroups all 2884 ERpositive/HER2negative samples were selected and a median split according to Ki67 expression was performed. In addition, all 106 ERpositive/HER2positive cases were also assigned to the Luminal B subtype according to this method (Hugh et al., 2009). Expression of the GABRP gene was measured across subtypes using probe set 205044_at. PAM50 classifier was used to define breast cancer subtypes from TCGA data.

Cell Lines HCC1143 cells were maintained in RPMI 1640 high-glucose medium with glutamate (ATCC), SK-BR-3 in McCoy's 5A (Gibco) while MDA-MB-468, MDA-MB-231, BT-474 and BT-549 cell lines were maintained in RPMI 1640 medium with glutamate (Gibco). All media were supplemented with 10% fetal bovine serum (FBS; Gibco), 100 units/ml penicillin, and 100 µg/ml streptomycin.

Immunoblotting PVDF membranes were blocked with 2% BSA in 10 mM Tris-HCl,50 mM NaCl, 0.1% Tween 20, pH 7.4 (TBST), followed by overnight incubation with intracellular domain (ICD)-binding anti-GABRP, ERBB2, $Na^{+}K^{+}$ATPase (Santa Cruz Biotechnology), extracellular domain (ECD)-binding GABRP (Abcam), phospho-AKT (Cell Signaling Technology) or LRP8 (Sigma) primary antibodies, diluted 1:5000 to 1:20000 in TBST/2% BSA. Membranes were washed five times with TBST, incubated with horseradish peroxide (HRP)-conjugated secondary antibodies in TBST/2% BSA for 1h, rinsed with TBST, and detected by chemiluminescence (SuperSignal West Pico Chemiluminescent Substrate; Pierce). Actin-HRP antibody (Santacruz Biotech) was used to measure actin level as a loading control for each lane.

Immunohistochemistry Briefly, slides were deparaffinized using xylene and rehydrated to distilled water. Endogenous peroxidase was quenched with hydrogen peroxide, and 1:75 dilution of primary antibody against GABRP was used. The slides were then washed with 10

mmol/L Tris-HCl, 50 mmol/L NaCl, 0.1% Tween 20, pH 7.4 (TBST) followed by incubation with horseradish peroxide-conjugated secondary antibody. Immunoreactivity was detected with the peroxidase-based Envision+ system (Dako). Diaminobenzidine (DAB) was used to detect the antibody complex (Dako). The slides were subsequently washed, counterstained with hematoxylin, dehydrated, cleared and coverslipped with resinous mounting media. Yale index breast cancer TMA (YTMA279-18) containing breast cancer tumors and cell lines including MDA-MB-468 and SK-BR3, was simultaneously stained using the same antibody. TMAs were scanned to create bright field digital images using the ScanScope CS (Aperio, Vista, CA). All digital images were viewed in ImageScope by a breast pathologist (V.P.) that scored tumor epithelial cells as a percentage of cells with GABRP signal. GABRP positivity threshold was set at >1% with minimum number of 100 tumor cells from each case on TMA. Areas with necrosis or inadequate quality of tissue/staining were excluded from scoring.

Flowcytometry

For this QuantiBRITE PE flow-cytometric analysis, isotype control IgG-PE antibody (R&D Systems) and ECD binding GABRP antibody conjugated to phycoerythrin (PE) were used. ECD binding GABRP (Abcam) was custom-conjugated to phycoerythrin (PE) in 1:1 ratio (Affymetrix eBiosciences). QuantiBRITE beads labeled with different PE levels were used to generate the standard curve for florescent intensity versus the number of PE molecules per bead. Mean number of GABRP receptors on cell surface was estimated by PE florescence intensity, as antibody binding capacity (ABC), following the manufacturers' protocol. Briefly, QuantiBRITE PE tube containing lyophilized pellet of beads conjugated with four levels of PE was ran on LSR-II flowcytometer to collect 10,000 events. Singlets were gated on the FSC-H vs SSC plot, and the singlet bead population was analyzed using a histogram plot of FL-2 axis in linear values. Log10 for lot-specific PE/bead values versus Log10 of geometric means for four bead peaks were plotted. Equation $y=mx+c$ where x equals Log10 PE molecules per bead and y equals Log10 fluorescence was used to calculate the slope,

intercept and correlation coefficient. To determine GABA antibodies bound per cell (ABC) for 5 cell lines, cellular assay samples were ran using the same instrumental settings and PE molecules per cell were calculated. Unstained cells and IgG-PE were used as negative controls. Similarly, surface GABRP was examined using Fab#1 in Figure 5 and Supplementary Figure 5. Briefly, $2.5X10^5$ cells/sample were trypsinized and washed with PBS, and fixed with dropwise addition of neutral buffered formalin (formaldehyde 3.7% w/v +methyl alcohol <1% v/v) for 20 min, followed by blocking with 2% BSA/PBS for 5 min and incubation with 10nM Fab in 2% BSA/PBS at 4^oC for 30 min. Since the Fab contains the FLAG tag, and anti-FLAG-PE (PE anti-DYKDDDDK Tag) antibody (BioLegend) was used for Fab detection on LSR-II flowcytometer.

T7 Endonuclease assay

Briefly, single guide RNAs (sgRNAs) were designed using CHOPCHOP (https://chopchop.rc.fas.harvard.edu/) and cloned into LentiCRISPRv2 (Addgene #52961). sgRNA sequences were GACCGGAACGATCTCGCGTA for scrambled (Vector Control), and CGACGTTGAACTGACTCCCC and AGAGTCAGCGCTATCTGTAC for two knock-outs KO-1 and KO-2 respectively. 1.5 μg lentiviral plasmid, 1 μg psPAX2, and 0.5 μg pMD2.G were transfected into 293T cells in 6-well plates using Lip2000. 48 hours after transfection, lentivirus-containing media were collected, filtered through a 0.45 μm filter, and used to infect cells. After infecting for 24 hours, cells were incubated with fresh media containing 1mg/ml of puromycin.

Genomic DNA was extracted using the DNeasy blood and tissue kit (Qiagen) following the manufacturer's protocol. The genomic region surrounding the target sites for each guide sequence was PCR amplified with Phusion polymerase (NEB) with the following program: preheat at 98°C for 60 s, 35 cycles of 3-step amplification (98°C for 15 s, 62°C for 15 s, 72°C for 30 s) and final extension at 72°C for 60 s. A total of 200 ng of the purified PCR products were

mixed with Buffer 2 (NEB) and ultrapure water to a final volume of 19 µl. Hybridization reactions were performed with the following program: 95°C for 5 min; ramp down to 85°C at −2°C/s; ramp down to 25°C at −0.1°C/s. Then 1 µl T7 endonuclease I (NEB) was added and the mixture was incubated at 37°C for 1 h. A total of 2 µl of 0.25M ethylenediaminetetraacetic acid was added to stop the reaction followed with gel electrophoresis on a 2% agarose gel. The primers for amplifying the region flanking GABRP sgRNA targeting site were as follows:

GABRP KO-1-F: TCTGTAGGAATGTCACTGCTGG

GABRP KO-1-R:AGAGGATGACCTACCACCAAAA

GABRP KO-2-F: TCATGGTTGTGTTTCCATTCTT

GABRP KO-2-R: CCCCTTTAAACACACAGAGAGG

siRNA-mediated RNA-interference

GABRP siRNA oligonucleotides included siRNA duplexes that target exons 7, 8, and 10, respectively and an equal mix of these 3 oligonucleotides (siGABRP) was used to ensure the most consistent knock-down. An unrelated control siRNA pool (Ambion) that lacks identity with known gene targets was used as a siRNA control (siControl) for non-sequence specific effects. The cells were plated in 6-well plates at 3.5 x 10^5 per well and transfected with siRNA 24h later using HiPerFect reagent (Qiagen) according to the manufacturer’s protocol. Three, human GABRP siRNA oligonucleotides were purchased from Ambion (Austin, TX). These included siRNA duplexes that targeted exons 7, 8, and 10, respectively (Ambion SiRNA ID numbers 114753, 118964, and 114754 for) with "UU" overhangs and a 5´ phosphate on the antisense strand. An equal mix of these 3 oligonucleotides was used to ensure the most consistent knock-down effect. An unrelated control siRNA pool (Ambion) that lacks identity with known gene targets was used as a scrambled control for non-sequence specific effects. In brief, HiPerFect reagent was diluted with serum-free medium in two-thirds the transfection volume for 10 minutes. It was then added to the diluted siRNA (GABRP or nonspecific siRNA pool) and incubated at ambient temperature for 20 minutes.

The culture medium was aspirated from the cells, and the cells were washed with serum-free medium. The siRNA-HiPerFect complex was added drop wise to the cells and incubated at 37°C for 4-6 hours, at which time one-third volume medium with 30% FBS was added and the cells were incubated for an additional 20 hours (for a total incubation period of 24 hours). The cells were then ready to be collected for further experiments.

Stable siRNA transfection

For stable knockdown, plasmids were packaged as virus by using phoenix-packaging cells (Orbigen, San Diego, CA) by FuGene 6 (Roche, Indianapolis, IN) following manufacturer's protocols. Cells were infected with virus in the presence of 4μg/mL polybrene (Sigma, St. Louis, MO) for 6 hours and allowed to recover for 24 hours in fresh medium. Infected cells were selected with puromycin 1–3 μg/mL for 2 weeks. Individual cell clones were selected and screened by qPCR for knockdown efficiency. We produced GABRP shRNA encoding pRETRO-SUPER-GABRP plasmid. Briefly, the pSUPER-PURO vector (Oligoengine, Seattle,WA) was digested with BglIII and HindIII and annealed oligonucleotides were ligated into the vector (5'gatccccGAAAGGAGATGTGGTGAAGttcaagagaCTTCACCACATCTCCTT Ctttttggaaa3'). The 19 nucleotide GABRP target sequence at position 121 are indicated in capitals in the oligonucleotide sequence. To generate pRETRO-SUPER-PURO constructs, a selfinactivating murine stem cell virus (pMSCV) plasmid was used. The 3′ LTR of the pMSCV was inactivated by an internal (NheI XbaI) deletion to generate a selfinactivating virus. Upon integration to the genome of the virus produced from this vector, this self-inactivating virus is duplicated to the 5′ LTR to generate a provirus that lacks all enhancer-promoter activities. EcoRI- and XhoI-digested inserts from pSUPER, pSUPER-GABRP (containing the polymerase III promoter and the targeting inserts) were cloned into the same sites in the self-inactivating pMSCV viral construct to generate the corresponding pSUPER-RETRO-PURO constructs. A scramble insert (Catalog # 87863, Oligoengine, Seattle, WA)

was cloned as a control. The plasmids were transfected into phoenix packaging cells (Orbigen, San Diego, CA) by FuGene 6 (Roche, Indianapolis, IN) according to manufacturer's instructions. The viral supernatant was used for infection of cells after addition of 4μg/mL polybrene (Sigma, St. Louis, MO).

Soft agar assay

In a 6-well culture plate, base layer was formed by 1.6% low-gelling temperature agarose (Sigma Aldrich) mixed with 2X RPMI complete medium (20% FBS), while the top layer contained 0.6% agarose, 2X RPMI complete medium and $1X10^4$ cells per well. 1X RPMI complete media was added and replenished every 3 days. Colony spheres formed were fixed and stained with 0.1% crystal violet and 2.1% citric acid.

Tumor Growth

Single-cell suspensions with >95% viability were used for mammary orthotopic injections in 8 weeks old mice, and tumor xenograft growth was monitored over time. MDA-MB-468 tumor cells with stable knockdown of GABRP were harvested by trypsinization, washed, resuspended in Ca2+ and Mg2+ free HBSS, and diluted to the desired cell number. Briefly, 2×10^6 tumor cells in 0.1 mL of RPMI-1640 serum free medium with 50% Matrigel (BD Matrigel, BD Biosciences, San Jose, CA,USA) were orthotopically injected at the 4th pair mammary gland on each side. Tumor growth was monitored by palpation and the onset of tumors was noted. Tumor size was measured with digital calipers and tumor volume was calculated assuming an ellipsoid shape with the following equation: Tumor volume (mm^3) = (Length x $Width^2$) x π/6. The animals were euthanized 10 weeks after tumor cell inoculation. Representative data were obtained from five mice per experimental group and the entire experiment was repeated in three independent trials.

GABRP antibody-DM1 ADC Immunogen method uses succinimidyl-4-(N-maleimidomethyl)-cyclohexane-1-carboxylate (SMCC) as linker which introduces maleimido group on the antibody to enable linkage of the DM1 via a non-reducible thioether bond. This non-

cleavable linker is therefore released only intracellularly. In addition to the rabbit polyclonal GABRP antibody, rabbit polyclonal isotype control antibody (Abcam) was also conjugated simultaneously to DM1 using same method. ADCs were characterized by hydrophobic interaction, size exclusion and reversed phase chromatography, UV-vis spectrophotometry, and the concentration of free drug in ADC was limited to <5%.

GABRP Overexpression

Lentivirus carrying vector control or GABRP plasmids with CMV promoter and GFP and puromycin cassettes was directly purchased from Applied Biological Materials Inc. Briefly, MDA-MB-231 cells were infected with control or GABRP lentivirus alongwith 4mg/ml polybrene in complete RPMI medium in 6-well culture plates. After 24h, virus-containing medium was replaced with fresh complete RPMI medium containing 1mg/ml of puromycin for selection.

Supplementary Table and Figure Legends

Supplementary Table 1 681 overexpressed genes in TNBC versus non-TNBC ranked by fold expression in 10 datasets: MDACC, Wang, TOP, Paris, IPC, Boston_2, expO, SanFrancisco, Boston, and Pool of 40 public datasets.

Supplementary Table 2 Details of 40 pooled datasets.

Supplementary Table 3 Details of 4467 samples from 40 datasets. Expression data, respective subtype designations and links to complete Affymetrix data for each individual sample are provided.

Supplementary Figure 1 mRNA expression of GABRP, GPNMB and ERBB2 in normal (green) and cancer (red) tissues, with the number of cases for each tissue type indicated in brackets on x-ases. Box-plots were generated from medisapiens.com where transcription profiles from large number of studies have been collated.

Supplementary Figure 2 Vertical bars represent log2 expression of GABRP mRNA in each breast cancer cell line, determined from Cancer Cell Line Encyclopedia (CCLE) data.

Supplementary Figure 3 Histogram (left) indicates fluorescence levels for the specific lot of QuantiBRITE PE beads with four levels of PE, read by LSR-II flowcytometer. Number of PE molecules/bead (4 levels) was provided with the kit and are indicated for each bead-PE population. Standard curve (right) was generated by plotting log fluorescence obtained from histogram versus on y-axis log PE/bead on x-axis. Intercept and correlation coefficient is indicated below.

Supplementary Figure 4 Immunoflorescent detection of GABRP (green) in breast cancer cell lines grown in culture showed membrane and cytoplasmic localization of the protein. Magnification 20X. Cell nuclei were counter-stained with Hoechst (blue) dye.

Supplementary Figure 5

GABRP overexpression in MDA-MB-231 cells. GABRP mRNA in vector control, GABRP, and parent MDA-MB-231 cells was measured by qPCR (A). GABRP overexpression in MDA-MB-231 was confirmed with flow-cytometry (red histogram) vs control cells (black histogram) (B) and by Western blot analysis of two biological replicates of vector control and GABRP-overexpressing MDA-MB-231 cell lysates, with GAPDH serving as loading control (C). Growth curves for GABRP-overexpressing MDA-MB-231 cells. Cell proliferation of vector control and GABRP-overexpressing MDA-MB-231 was assessed by the CellTiter-Glo luminescent cell viability assay every day over a 5-day culture period. Data points fold change over day-0 mean luminescence per well ±SEM in each group. $^*P < 0.05$ by one-way ANOVA followed by the Newman–Keuls multiple comparison test. Brightfiled and immunoflorescent images of green florescent protein (GFP)-expressing vector control and GABRP MDA-MB-231 cell lines while under selection with 1ug/ml puromycin in culture. Magnification 20X (E).

Supplementary Figure 1

GABRP mRNA Expression in Human Tissues

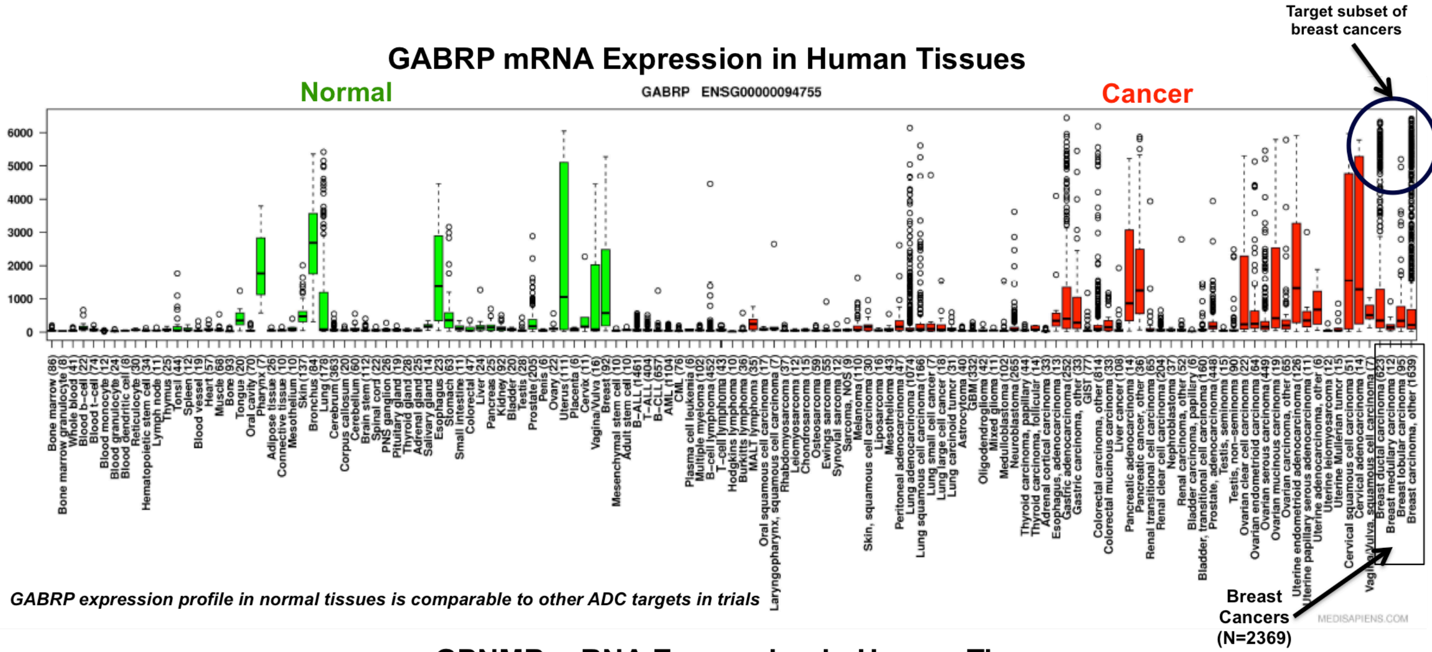

GABRP expression profile in normal tissues is comparable to other ADC targets in trials

GPNMB mRNA Expression in Human Tissues

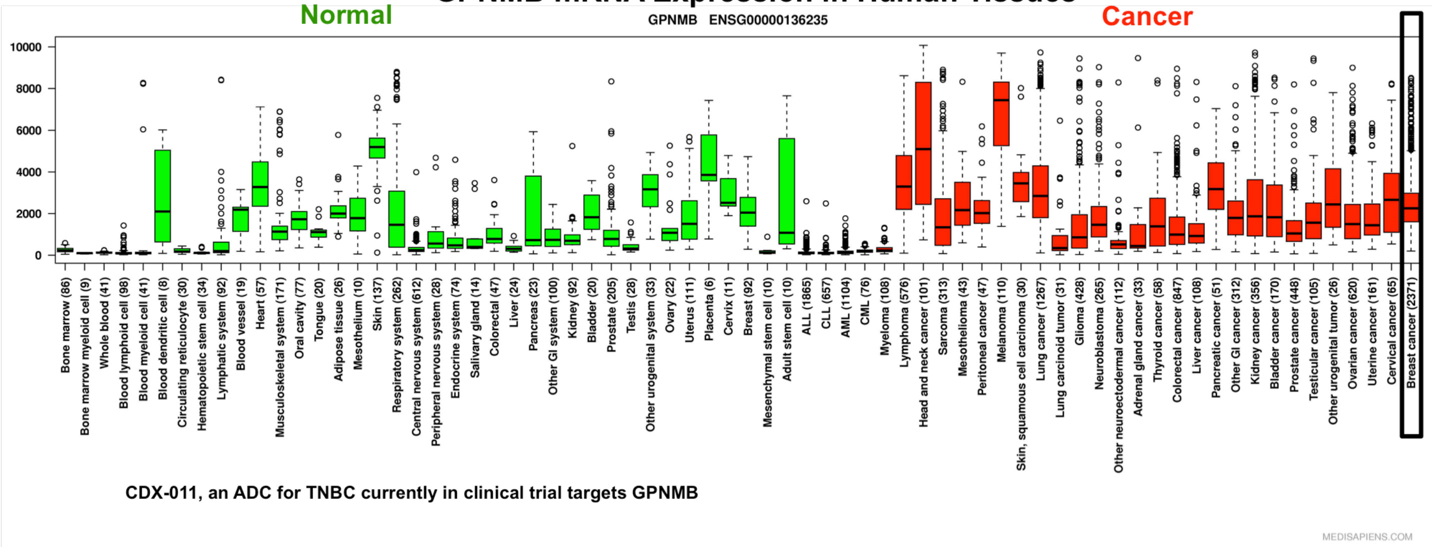

CDX-011, an ADC for TNBC currently in clinical trial targets GPNMB

ERBB2 mRNA Expression in Human Tissues

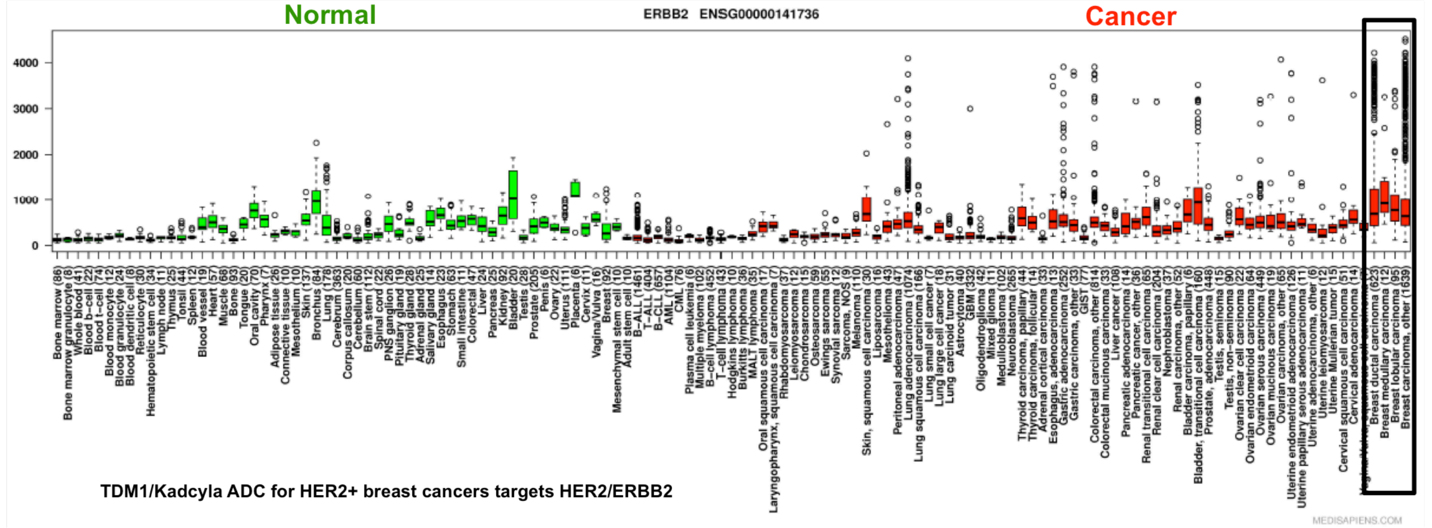

TDM1/Kadcyla ADC for HER2+ breast cancers targets HER2/ERBB2

mediasapies.com

Supplementary Figure 2

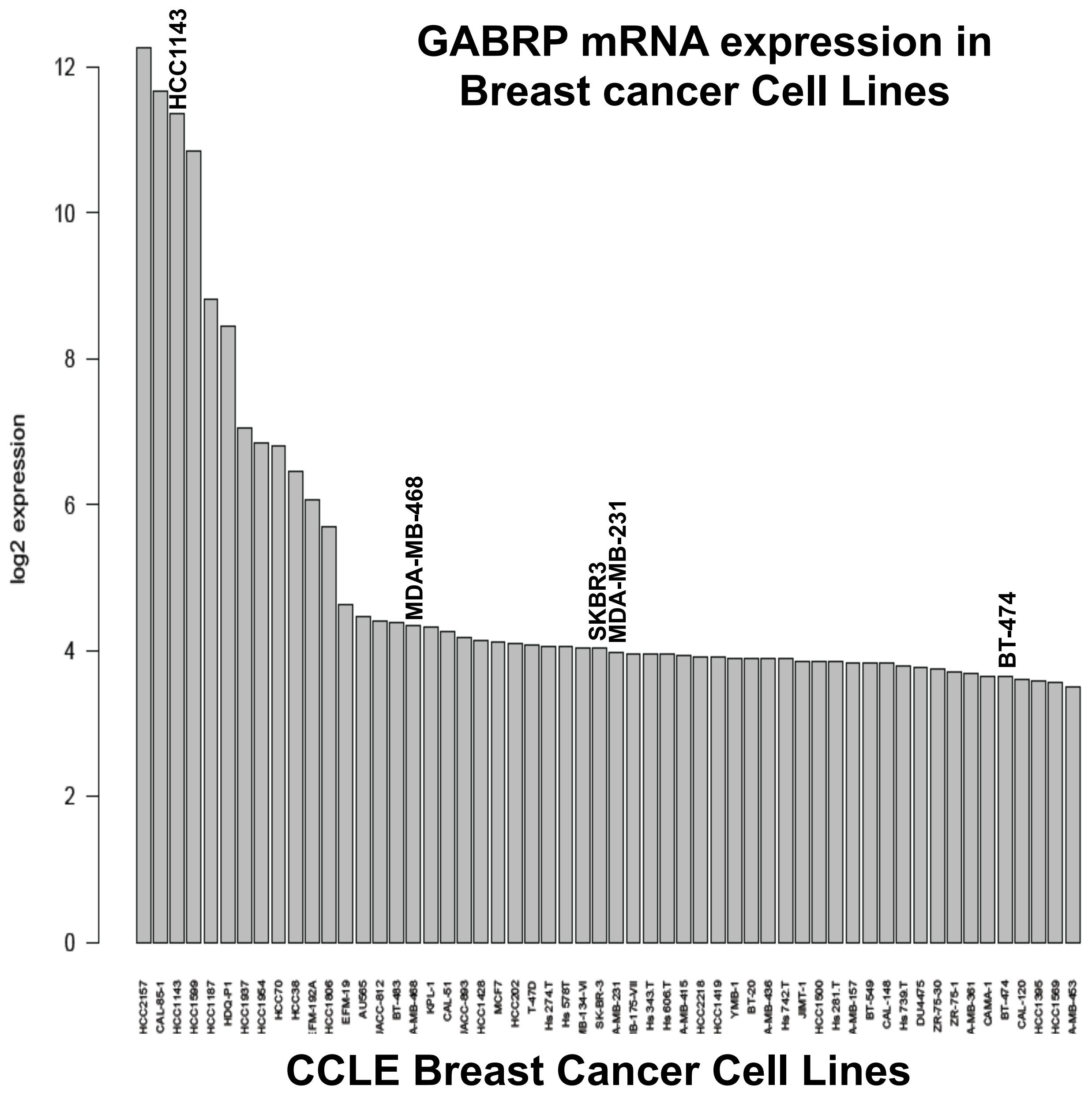

Supplementary Figure 3

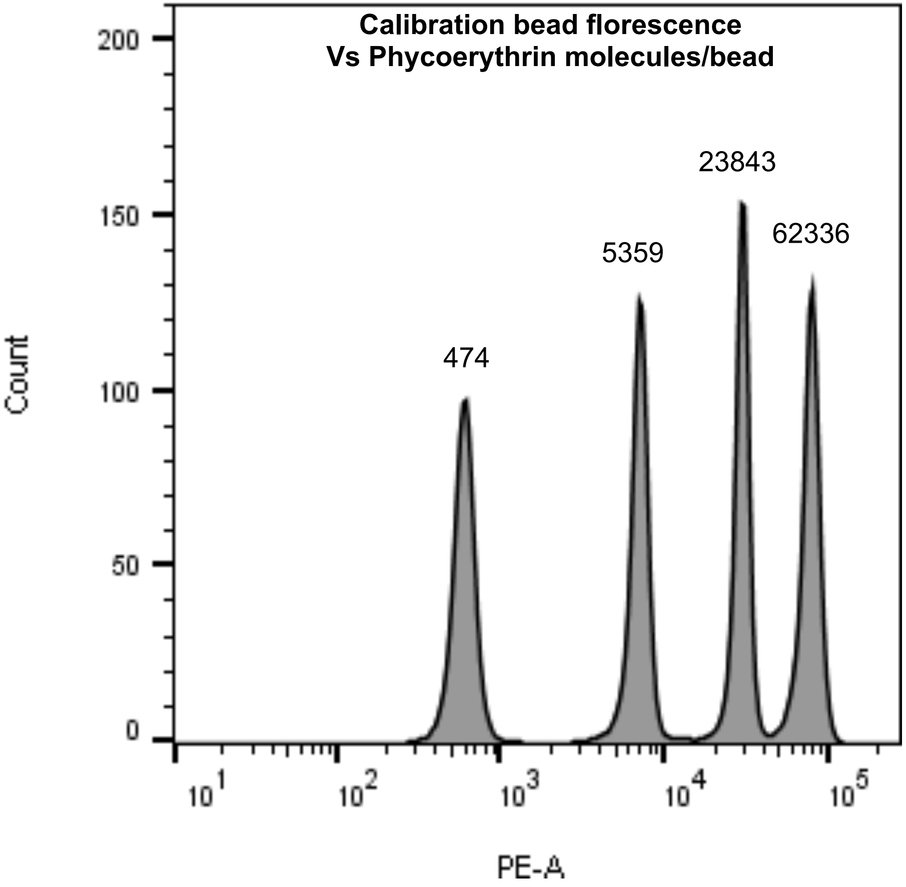

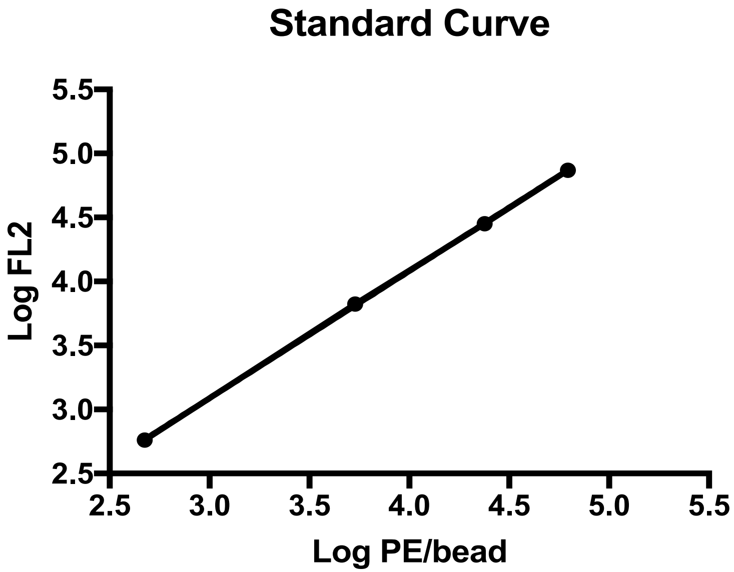

	Log FL2
Equation	Y = 0.993*X + 0.1092

	Log FL2
R square	0.9999

Supplementary Figure 4

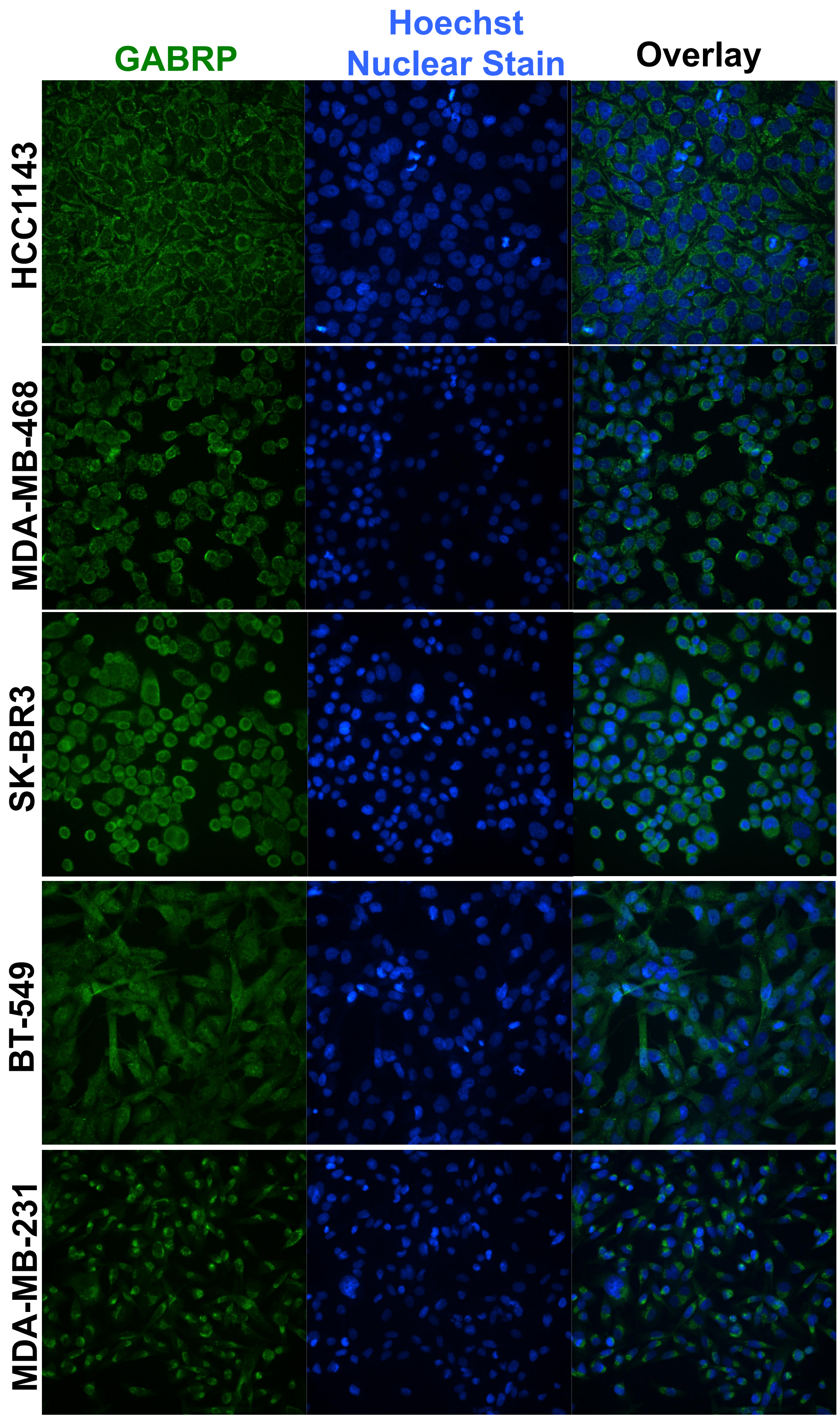

Supplementary Figure 5

GABRP Overexpressing MDA-MB-231 Cells

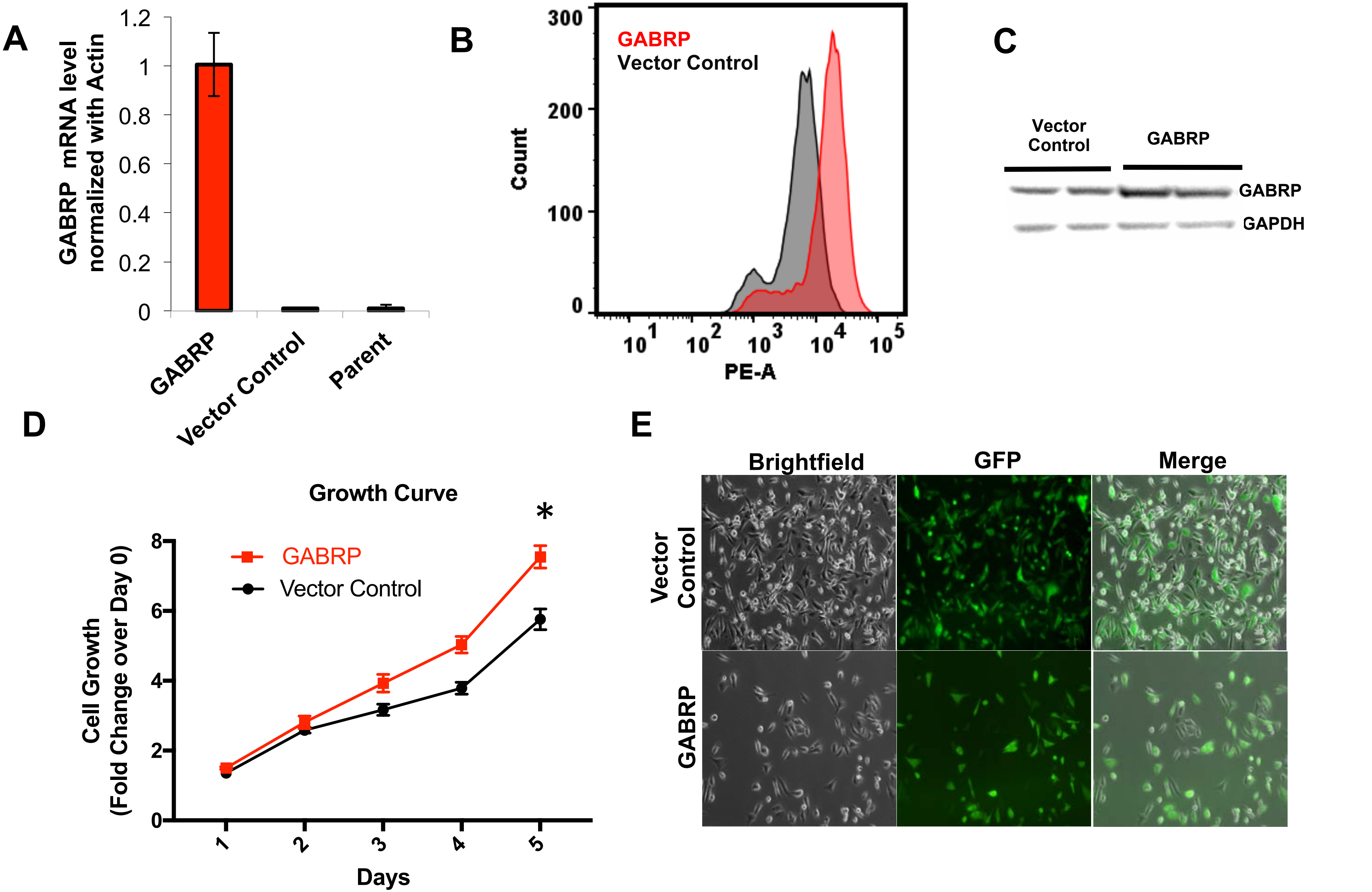

Supplementary Table 2: Datasets used in the study (n=40)

Datasets used in the study:

*Dataset **	*Data source*	*Number of samples*	*Reference (PubMed link)*	*Ref.*
Stockholm	GSE1456	159	http://www.ncbi.nlm.nih.gov/pubmed/16280042	1
EORTC	GSE1561	49	http://www.ncbi.nlm.nih.gov/pubmed/15897907	2
Rotterdam-EMC344	GSE2034, GSE5327	344	http://www.ncbi.nlm.nih.gov/pubmed/15721472, 17420468	3,4
expO	GSE2109	301	http://www.intgen.org/expo/	
New York	GSE2603	99	http://www.ncbi.nlm.nih.gov/pubmed/16049480	5
Oxford-Untreated	GSE2990 (n=61), GSE6532 (n=8)	69	http://www.ncbi.nlm.nih.gov/pubmed/16478745	6
Uppsala	GSE3494 (n=251), GSE6232 (n=5), GSE4922 (n=1), GSE2990 (n=1)	258	http://www.ncbi.nlm.nih.gov/pubmed/16141321,%2017079448	7,8
Boston	GSE3744	40	http://www.ncbi.nlm.nih.gov/pubmed/16473279	9
Signapore	GSE5364	183	http://www.ncbi.nlm.nih.gov/pubmed/18636107	10
Edinburgh	GSE5462	116	http://www.ncbi.nlm.nih.gov/pubmed/17885619	11
London	GSE6532	87	http://www.ncbi.nlm.nih.gov/pubmed/17401012	12
Oxford-Tamoxifen	GSE6532	109	http://www.ncbi.nlm.nih.gov/pubmed/16478745	6
Berlin	GSE6596	24	http://www.ncbi.nlm.nih.gov/pubmed/17410534	13
TransBIG	GSE7390	198	http://www.ncbi.nlm.nih.gov/pubmed/17545524	14
London-2	GSE9195	77	http://www.ncbi.nlm.nih.gov/pubmed/18498629	15
Tampa	GSE10780	39	http://www.ncbi.nlm.nih.gov/pubmed/19266279	16
Mainz	GSE11121	200	http://www.ncbi.nlm.nih.gov/pubmed/18593943	17
Veridex-Tam	GSE12093	136	http://www.ncbi.nlm.nih.gov/pubmed/18821012	18
Rotterdam-EMC204	GSE12276	204	http://www.ncbi.nlm.nih.gov/pubmed/19421193	19
Genentech	GSE12763	30	http://www.ncbi.nlm.nih.gov/pubmed/19567590	20
Paris	GSE13787	23	http://www.ncbi.nlm.nih.gov/pubmed/19055754	21
BIG1-98	GSE16391	55	http://www.ncbi.nlm.nih.gov/pubmed/19573224	14
TOP	GSE16446	120	http://www.ncbi.nlm.nih.gov/pubmed/21422418	22
MDA100	GSE16716	100	http://www.ncbi.nlm.nih.gov/pubmed/20064235	23
SET1	GSE17705	103	http://www.ncbi.nlm.nih.gov/pubmed/20697068	24
SET2	GSE17705	195	http://www.ncbi.nlm.nih.gov/pubmed/20697068	24
IPC_HER2	GSE17907	51	http://www.ncbi.nlm.nih.gov/pubmed/20932292	25
Seattle	GSE18728	21	http://www.ncbi.nlm.nih.gov/pubmed/20012355	26
Boston_Neo-Cisplatin	GSE18864	84	http://www.ncbi.nlm.nih.gov/pubmed/20100965	27
Boston_2	GSE19615	115	http://www.ncbi.nlm.nih.gov/pubmed/20098429	28
StLouis	GSE19697	24	http://www.ncbi.nlm.nih.gov/pubmed/19967557	29
Edinburgh	GSE20181	60	http://www.ncbi.nlm.nih.gov/pubmed/20697427	11
MAQC_add_GSE20194	GSE20194	45	http://www.ncbi.nlm.nih.gov/pubmed/20676074	30
MDA_139	GSE20271 (n=139 additional to MDA133, GSE16716, and GSE20194)	139	http://www.ncbi.nlm.nih.gov/pubmed/20829329	31
IPC	GSE21653	266	http://www.ncbi.nlm.nih.gov/pubmed/20490655	32
St-Cloud	GSE22035	43	http://www.ncbi.nlm.nih.gov/pubmed/21209903	33
Nashville	GSE22513	28	http://www.ncbi.nlm.nih.gov/pubmed/20068102	34
San Francisco	http://www.ebi.ac.uk/arrayexpress/experiments/E-TABM-158	118	http://www.ncbi.nlm.nih.gov/pubmed/17157792	35
Neo-Trastuzumab	https://array.nci.nih.gov/caarray/project/harri-00137	22	http://www.ncbi.nlm.nih.gov/pubmed/17317830	36
MDA133	http://bioinformatics.mdanderson.org/pubdata.html	133	http://www.ncbi.nlm.nih.gov/pubmed/16896004	37
TOTAL:		***4467***		

**** Remarks**:* The complete *TransBIG* dataset contains independent replicate samples from 19 patients of the *Uppsala* cohort and 22 patients of the *Oxford-Untreated* cohort. Datasets "*MAQC_add_GSE20194*" and "*MDA_139*" contain only the subsets of 45 and 139 nonredundant samples from the GEO series GSE20194 and GSE20271, respectively, which are not already covered by the *MDA133* and *MDA100* datasets.

References to Supplementary Table 2

1. Pawitan, Y. *et al.* Gene expression profiling spares early breast cancer patients from adjuvant therapy: derived and validated in two population-based cohorts. *Breast Cancer Res* **7**, R953-64 (2005).
2. Farmer, P. *et al.* Identification of molecular apocrine breast tumours by microarray analysis. *Oncogene* **24**, 4660–4671 (2005).
3. Wang, Y. *et al.* Gene-expression profiles to predict distant metastasis of lymph-node-negative primary breast cancer. *Lancet* **365**, 671–679 (2005).
4. Minn, A. J. *et al.* Genes that mediate breast cancer metastasis to lung. *Nature* **436**, 518–524 (2005).
5. Minn, A. J. *et al.* Lung metastasis genes couple breast tumor size and metastatic spread. *Proc Natl Acad Sci U S A* **104**, 6740–6745 (2007).
6. Sotiriou, C. *et al.* Gene expression profiling in breast cancer: understanding the molecular basis of histologic grade to improve prognosis. *J Natl Cancer Inst* **98**, 262–272 (2006).
7. Miller, L. D. *et al.* An expression signature for p53 status in human breast cancer predicts mutation status, transcriptional effects, and patient survival. *Proc Natl Acad Sci U S A* **102**, 13550–13555 (2005).
8. Ivshina, A. V. *et al.* Genetic reclassification of histologic grade delineates new clinical subtypes of breast cancer. *Cancer Res* **66**, 10292–10301 (2006).
9. Richardson, A. L. *et al.* X chromosomal abnormalities in basal-like human breast cancer. *Cancer Cell* **9**, 121–132 (2006).
10. Yu, K. *et al.* A precisely regulated gene expression cassette potently modulates metastasis and survival in multiple solid cancers. *PLoS Genet* **4**, e1000129 (2008).
11. Miller, W. R., Larionov, A., Anderson, T. J., Evans, D. B. & Dixon, J. M. Sequential changes in gene expression profiles in breast cancers during treatment with the aromatase inhibitor, letrozole. *Pharmacogenomics J* **12**, 10–21 (2012).
12. Loi, S. *et al.* Definition of clinically distinct molecular subtypes in estrogen receptor-positive breast carcinomas through genomic grade. *J Clin Oncol* **25**, 1239–1246 (2007).
13. Klein, A. *et al.* Comparison of gene expression data from human and mouse breast cancers: identification of a conserved breast tumor gene set. *Int J Cancer* **121**, 683–688 (2007).
14. Desmedt, C. *et al.* The Gene expression Grade Index: a potential predictor of relapse for endocrine-treated breast cancer patients in the BIG 1-98 trial. *BMC Med Genomics* **2**, 40 (2009).
15. Loi, S. *et al.* Predicting prognosis using molecular profiling in estrogen receptor-positive breast cancer treated with tamoxifen. *BMC Genomics* **9**, 239 (2008).
16. Chen, D.-T. *et al.* Proliferative genes dominate malignancy-risk gene signature in histologically-normal breast tissue. *Breast Cancer Res Treat* **119**, 335–346 (2010).
17. Schmidt, M. *et al.* The humoral immune system has a key prognostic impact in node-negative breast cancer. *Cancer Res.* **68**, 5405–5413 (2008).
18. Zhang, Y. *et al.* The 76-gene signature defines high-risk patients that benefit from adjuvant tamoxifen therapy. *Breast Cancer Res Treat* **116**, 303–309 (2009).
19. Bos, P. D. *et al.* Genes that mediate breast cancer metastasis to the brain. *Nature* **459**, 1005–1009 (2009).
20. Hoeflich, K. P. *et al.* In vivo antitumor activity of MEK and phosphatidylinositol 3-kinase inhibitors in basal-like breast cancer models. *Clin Cancer Res* **15**, 4649–4664 (2009).
21. Marty, B. *et al.* Frequent PTEN genomic alterations and activated phosphatidylinositol 3-kinase pathway in basal-like breast cancer cells. *Breast Cancer Res.* **10**, R101 (2008).
22. Desmedt, C. *et al.* Multifactorial approach to predicting resistance to anthracyclines. *J Clin Oncol* **29**, 1578–1586 (2011).
23. Popovici, V. *et al.* Effect of training-sample size and classification difficulty on the accuracy of genomic predictors. *Breast Cancer Res* **12**, R5 (2010).
24. Symmans, W. F. *et al.* Genomic index of sensitivity to endocrine therapy for breast cancer. *J Clin Oncol* **28**, 4111–4119 (2010).
25. Sircoulomb, F. *et al.* Genome profiling of ERBB2-amplified breast cancers. *BMC Cancer* **10**, 539 (2010).
26. Korde, L. A. *et al.* Gene expression pathway analysis to predict response to neoadjuvant docetaxel and capecitabine for breast cancer. *Breast Cancer Res Treat* **119**, 685–699 (2010).
27. Silver, D. P. *et al.* Efficacy of neoadjuvant Cisplatin in triple-negative breast cancer. *J Clin Oncol* **28**, 1145–1153 (2010).
28. Li, Y. *et al.* Amplification of LAPTM4B and YWHAZ contributes to chemotherapy resistance and recurrence of breast cancer. *Nat Med* **16**, 214–218 (2010).
29. Lin, Y. *et al.* A gene expression signature that predicts the therapeutic response of the basal-like breast cancer to neoadjuvant chemotherapy. *Breast Cancer Res Treat* **123**, 691–699 (2010).
30. Shi, L. *et al.* The MicroArray Quality Control (MAQC)-II study of common practices for the development and validation of microarray-based predictive models. *Nat Biotechnol* **28**, 827–838 (2010).
31. Tabchy, A. *et al.* Evaluation of a 30-gene paclitaxel, fluorouracil, doxorubicin, and cyclophosphamide chemotherapy response predictor in a multicenter randomized trial in breast cancer. *Clin Cancer Res* **16**, 5351–5361 (2010).
32. Sabatier, R. *et al.* A gene expression signature identifies two prognostic subgroups of basal breast cancer. *Breast Cancer Res. Treat.* **126**, 407–420 (2011).
33. Cizkova, M. *et al.* Gene expression profiling reveals new aspects of PIK3CA mutation in ERalpha-positive breast cancer: major implication of the Wnt signaling pathway. *PLoS ONE* **5**, e15647 (2010).
34. Bauer, J. A. *et al.* Identification of markers of taxane sensitivity using proteomic and genomic analyses of breast tumors from patients receiving neoadjuvant paclitaxel and radiation. *Clin Cancer Res* **16**, 681–690 (2010).
35. Chin, K. *et al.* Genomic and transcriptional aberrations linked to breast cancer pathophysiologies. *Cancer Cell* **10**, 529–541 (2006).
36. Harris, L. N. *et al.* Predictors of resistance to preoperative trastuzumab and vinorelbine for HER2-positive early breast cancer. *Clin Cancer Res* **13**, 1198–1207 (2007).
37. Hess, K. R. *et al.* Pharmacogenomic predictor of sensitivity to preoperative chemotherapy with paclitaxel and fluorouracil, doxorubicin, and cyclophosphamide in breast cancer. *J Clin Oncol* **24**, 4236–4244 (2006).
